# Giant saphenous vein graft aneurysm and associated constrictive pericarditis in a patient with prior CABG

**DOI:** 10.21542/gcsp.2025.57

**Published:** 2025-10-31

**Authors:** Emily L. Larson, Binuri Hapuarachchy, Hamza Aziz

**Affiliations:** Division of Cardiac Surgery, Department of Surgery, Johns Hopkins School of Medicine, 1800 Orleans St, Baltimore, MD 21287

## Abstract

Background: Saphenous vein graft aneurysms (SVGA) are a rare but significant complication following coronary artery bypass grafting. They can lead to life-threatening conditions including rupture, fistula formation, and compression of adjacent thoracic structures. Current consensus calls for intervention, either surgically or percutaneously, with no agreed upon threshold for observation measures only. This case describes the clinical course of a patient with a known SVGA a decade prior to their current presentation with constrictive pericarditis.

Case presentation: The patient developed a large SVGA after undergoing coronary artery bypass grafting. The SVGA was monitored for over a decade without intervention. The patient then developed worsening symptoms of volume overload and was found to have developed a constrictive left-sided pericarditis due to the SVGA compressing left-ventricular inflow. Two pericardiectomies were performed, with debridement of the SVGA and excision of calcified pericardial plaques. Although the constriction was addressed, the patient was unable to recover and ultimately died.

Discussion: This report characterizes an SVGA causing constrictive pericarditis following coronary artery bypass grafting. This case demonstrates the need for appropriate and timely intervention in managing vein graft aneurysms and preventing fatal complications. We recommend annual echocardiography with dedicated evaluation for constriction to guide the need for intervention in patients with SVGA.

## Introduction

Saphenous vein graft aneurysms (SVGA) are a rare but significant complication following coronary artery bypass grafting (CABG). The most commonly used grafts for CABG are the left internal mammary artery and lower extremity saphenous veins^1^. SVGAs are a rare complication, occurring when the grafted vessel expands to more than 1.5 times its original size^2^, and have a reported incidence of 0.07%^3^. This is likely an underestimation given their frequent incidental diagnosis. They are most commonly observed in those who have undergone CABG one to two decades prior to the finding^4^.

Memon et al. report that mild dilatation of saphenous vein grafts can be expected and was observed in 14% of cases at 5–7 years after surgery in their cohort^5^, whereas the size of identified aneurysms tend to far exceed the diagnostic threshold^6^. The pathogenesis often involves pseudoaneurysm formation at the body or anastomotic sites of the graft, with intraluminal thrombi common^7^. These aneurysms can present variably, from being asymptomatic to causing life-threatening complications. Previous reports have described cases of rupture, fistula formation, and compression of neighboring cardiac and vascular structures leading to hemodynamic compromise^8–10^.

Current proposed guidelines consider factors such as symptoms, SVGA size, surgical risk, myocardial viability, and SVGA character to guide decisions on intervention^11^. Typically, intervention options for SVGAs include surgical or percutaneous approaches^3^. Surgical treatment involves excision of the aneurysm with direct revascularization of the coronary target vessel or interposition grafting. Percutaneous interventions utilize devices such as covered stents, which can be advantageous by avoiding risks associated with repeat sternotomy^12^. The consequences of non-intervention can be severe, with the in-hospital mortality associated with giant SVGAs described by Almanaseer et al. to be 15.7%^13^. Therefore, timely diagnosis and intervention are crucial to prevent adverse outcomes in patients with SVGA.

### Case report

**Figure 1.**
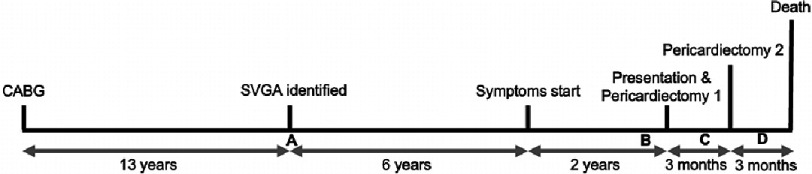
Timeline of course of illness for patient with saphenous vein graft aneurysm (SVGA) after coronary artery bypass grafting (CABG).

**Figure 2.**
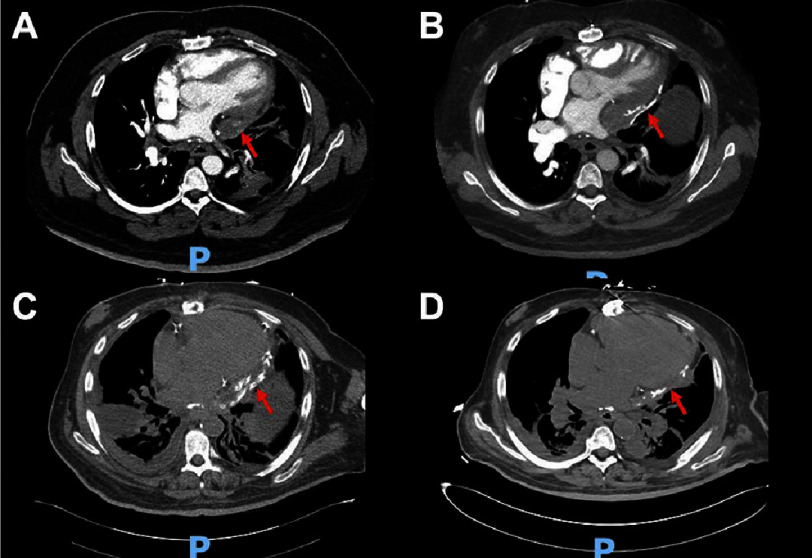
Computed tomography imaging of patient with saphenous vein graft aneurysm from time of diagnosis (A), after symptom onset (B), after first pericardiectomy (C), and after second pericardiectomy (D). The location of the saphenous vein graft aneurysm noted with red arrows. Letters are also noted in Figure 1 timeline.

The patient was a 57-year-old man with past medical history including coronary artery disease, heart failure with preserved ejection fraction (HFpEF), type II diabetes mellitus, hypertension, hyperlipidemia, chronic back pain, chronic kidney disease, and cirrhosis with recurrent ascites. He underwent CABG 21 years earlier, with grafts from the left internal mammary artery to the left anterior descending artery, right internal mammary to the posterior descending artery, and saphenous vein grafts to obtuse marginal 1 and obtuse marginal 2. The CABG was complicated by left diaphragm paralysis. A calcified pericardial mass compressing the left atrium and ventricle, thought to be a thrombosed SVGA, was subsequently identified 8 years prior to presentation (Figure 1, Figure 2A). It was considered stable and asymptomatic and did not undergo further evaluation or treatment.

**Figure 3.**
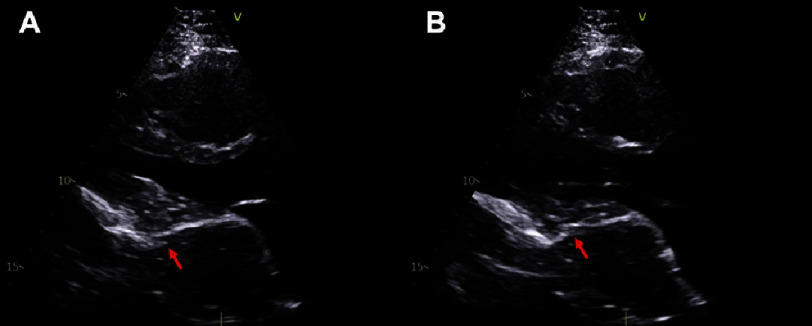
Transthoracic echocardiography of patient with saphenous vein graft aneurysm after symptom onset before first pericardiectomy with exaggerated septal bounce. Systole (A) and diastole (B). Red arrows denote the location of the saphenous vein graft aneurysm.

Over the two years prior to evaluation by the cardiothoracic surgery team, the patient had multiple admissions for management of HFpEF, with worsening dyspnea, fatigue, lower extremity edema, and ascites. During these hospitalizations, computed tomography (CT) imaging continued to show the SVGA (Figure 2B), which was still considered to be largely noncontributory to the patient’s presentation. It was echocardiographic findings concerning for constrictive pericarditis, including exaggerated mitral inflow respiratory variation and increased septal bounce (Figure 3), that increased the suspicion that the SVGA may be clinically pathologic. Right and left cardiac catheterizations were performed and showed elevated right atrial pressure (24 mmHg) associated with elevated pulmonary capillary wedge pressure (31 mmHg), elevated mean pulmonary artery pressure (51 mmHg) consistent with pulmonary hypertension, and a normal cardiac index (2.27 L/minute/m^2^). All grafts besides the left internal mammary to left anterior descending artery were occluded. Based on these findings, it was thought that the thrombosed SVGA had led to a left-sided pericarditis and left ventricular inflow obstruction causing the patient’s heart failure symptoms, pulmonary hypertension, and cardiogenic cirrhosis.

The patient was evaluated for pericardiectomy by cardiothoracic surgery, with several important considerations. Technically, the position of the SVGA over the left side of the heart was challenging, as exposing it required dissecting the LIMA pedicle away from a scarred and thickened pericardium. Thus, multiple approaches were considered, including sternotomy, hemiclamshell incision, or anterolateral thoracotomy, ultimately electing to perform a median sternotomy. Second, the patient unfortunately had a fall prior to presentation required prolonged cervical spine immobilization, so his operation was postponed until around four months later.

After preoperative volume optimization, the patient underwent midline sternotomy. After sufficient dissection, the patient was cannulated through the aorta and right femoral vein, and cardiopulmonary bypass was initiated. The LIMA was identified and the pericardium incised distally and laterally to allow the heart to medialize.

Next, the thrombosed SVGA was identified; it was large and required extensive debridement. The SVGA was ligated with clips proximally. Complete excision of the SVGA was attempted but unsuccessful for multiple reasons. First, there was no clear plane of dissection for the SVGA, which made it difficult to distinguish SVGA from surrounding tissue. Second, the LIMA was intact and in close proximity to the SVGA, so caution was taken to avoid injury to the LIMA. Finally, the heart was extremely adherent posteriorly. Near the mitral annulus and left atrial appendage, there was an area of very calcified plaque; when dissected, there was bleeding from a coronary vessel, which was repaired by tacking down the plaque to achieve hemostasis. For these reasons, the SVGA was debrided as much as was safely possible but was not fully excised. A degloving injury of the inferior border of the heart during this dissection and mobilization of the heart. It was repaired with pledgeted sutures with hemostasis achieved.

Dense, calcified adhesions along the diaphragmatic and lateral border of the heart were taken down. Bleeding near the inferior vena cava was identified, and the denuded tissue was repaired with a bovine pericardial patch. Some of the pericardium remained densely adhered to the lung and was unable to be safely freed.

Cardiopulmonary bypass was weaned after 170 min, and the patient was evaluated for ongoing bleeding. However, the bleeding was dark and venous appearing without any repairable sites identified. Multiple hemostatic agents were applied and blood products given. The patient continued to bleed and required multiple vasopressors for hemodynamic support. With low mean arterial pressures and a high vasopressor requirement, an intra-aortic balloon pump was placed and the chest packed and left open. The patient was brought to the intensive care unit (ICU).

The patient returned to the operating room that night after his chest tube rapidly output rapidly increased to 1.5 L/hour. The patient was again placed on cardiopulmonary bypass for 35 min to repair an injured epicardial vessel with overlying calcified plaque. The patient then again returned to the ICU with an open chest. The patient returned to the operating room the next day for an uneventful chest closure.

The postoperative course was complicated by respiratory failure requiring tracheostomy, dysphagia requiring gastrostomy tube, and ascites requiring multiple paracenteses. The patient was discharged to a rehabilitation facility after around one month in the hospital. Unfortunately, the patient returned within a week with recurrent volume overload. Repeat echocardiography and CT imaging showed continued constriction around the heart (Figure 2C).

Three months from the initial pericardiectomy, a second pericardiectomy was performed to address this residual constriction. Single-lung ventilation was used to enable a left lower thoracotomy at the 8th intercostal space. The left diaphragm was plicated, and a 5 × 7 centimeter plaque was removed from the posterolateral pericardium. His postoperative course was complicated by a right-sided pleural effusion requiring a decortication. He also developed a lymphocele at the site of venous cannulation in the groin during the index operation requiring evacuation.

Despite the repeat pericardiectomy and relief of the constriction (Figure 2D), the patient continued to deteriorate. While admitted for around three more months, he had worsening mental status and volume overload. He had a cardiopulmonary arrest with polymorphic ventricular tachycardia but obtained return of spontaneous circulation. Unfortunately, the patient had another cardiopulmonary arrest four days later, this time with pulseless electrical activity. He was unable to be resuscitated and died.

## Discussion

This report details an SVGA compressing the left ventricular in-flow and causing constrictive left-sided pericarditis. As in this case, especially when complicated by constrictive pericarditis or other pathology, SVGAs can pose a significant threat to hemodynamic stability and forward flow. In this patient, given the existing knowledge of the aneurysm starting a decade prior and concurrent thrombosis, surgical intervention prior to their acute deterioration may have prevented morbidity and mortality.

The underlying mechanism of the observed SVGA and associated constrictive pericarditis is not established. SVGAs have been observed to cause symptoms from mass effect^14,15^, but there are not reports of associated local pericarditis. While there are reports of constrictive pericarditis after CABG^16^, especially in the setting of post-cardiotomy syndrome, a localized constrictive pericarditis at the site of an SVGA as seen here has not been previously reported. Given the anatomic proximity of the SVGA and area of constrictive pericarditis, we hypothesize that the development of the local constrictive pericarditis near the SVGA was due to a combination of local inflammation and mass effect from the SVGA causing fibrosis and scarring.

The SVGA described here was monitored for nearly a decade without intervention. At the time, it was thought the risk of reoperation outweighed the benefit of correction in an asymptomatic patient. In retrospect, earlier intervention may have saved this patient some of the morbidity and mortality that occurred when he presented in an already decompensated state for surgery. Based on current consensus, there does not appear to be a safe threshold for monitoring SVGA without intervention^2^. It has been reported that 20 mm aneurysms can be correlated with a 33.3% rate of complications while aneurysms greater than 100 mm in size have a complication rate of up to 69.2%^2^. A proposed guideline for management of SVGAs includes inputs of symptom presence, SVGA size, surgical risk, myocardial viability, and SVGA character to guide decisions on intervention^11^. Based on this patient’s course, our practice for patients with SVGAs will now include annual echocardiography to evaluate for constriction with a low threshold for intervention if signs of constriction are present.

The patient’s symptoms persisted after the initial pericardiectomy despite the intervention. Overall, the patient’s condition had significantly progressed at the time of presentation with hepatic congestion and cardiac cirrhosis. Thus, interventions at this stage likely were not sufficient to reverse the underlying pathophysiology. While the resection of the SVGA was incomplete due to anatomic factors, the mass effect of the SVGA was felt to have been relieved. There was some pericardium unable to be excised due to its adherence to the lung, which may have contributed to symptom persistence. However, even after pericardiectomy of the residual pericardium, the patient remained volume overloaded likely due to a combination of his baseline comorbidities and residual constriction and ultimately detiorated.

While percutaneous approaches for SVGA intervention exist, in the event that an SVGA is complicated by thrombosis, compression of adjacent cardiac structures, fistula formation, or rupture, surgical intervention may be the only feasible option^2^. This approach has shown satisfactory mid- to long-term outcomes, with survival rates of 83% at 5 years and 72% at 10 years post-repair^7^. For this specific patient, there was no clear lumen in the SVGA, which made him a poor candidate for percutaneous intervention. Medical management of the constrictive pericarditis with anti-interleukin-1 therapy may also be a reasonable first-line treatment before pericardiectomy for patients with constrictive pericarditis associated with SVGA^17^.

Taken together, given the favorable outcomes of patients following intervention for SVGAs and their potentially fatal consequences, caution and earlier surgical management may prevent such adverse outcomes as seen here. We recommend annual surveillance with echocardiography with dedicated evaluation of constriction to guide decision on intervention in patients with SVGAs.

## What have we learned?

This case describes the potential effects on morbidity and subsequent mortality of a known saphenous vein graft aneurysm in a patient with a prior CABG, whose presentation was complicated by constrictive pericarditis. This serves as a cautionary tale against non-intervention in saphenous vein graft aneurysms, a rare but significant complication of CABG and highlights the importance of ongoing surveillance for constrictive pathophysiology.

## Author contributions

EL: Manuscript writing, literature review

BH: Manuscript writing, literature review

HA: Conceptualization, critical review of manuscript

## Conflicts of interest

The authors have no financial conflicts of interest to disclose and received no funding for this work.
